# Surface temperatures in New York City: Geospatial data enables the accurate prediction of radiative heat transfer

**DOI:** 10.1038/s41598-018-19846-5

**Published:** 2018-02-02

**Authors:** Masoud Ghandehari, Thorsten Emig, Milad Aghamohamadnia

**Affiliations:** 1 New York University, Tandon School of Engineering, and the Center for Urban Science and Progress, Brooklyn, NY USA; 2 Massachusetts Institute of Technology, MultiScale Materials Science for Energy and Environment, Joint MIT-CNRS Laboratory UMI 3466, Cambridge, Massachusetts 02139 USA; 3 Laboratoire de Physique Théorique et Modèles Statistiques, CNRS UMR 8626, Université Paris-Sud, Université Paris-Saclay, 91405 Orsay, cedex France

## Abstract

Despite decades of research seeking to derive the urban energy budget, the dynamics of thermal exchange in the densely constructed environment is not yet well understood. Using New York City as a study site, we present a novel hybrid experimental-computational approach for a better understanding of the radiative heat transfer in complex urban environments. The aim of this work is to contribute to the calculation of the urban energy budget, particularly the stored energy. We will focus our attention on surface thermal radiation. Improved understanding of urban thermodynamics incorporating the interaction of various bodies, particularly in high rise cities, will have implications on energy conservation at the building scale, and for human health and comfort at the urban scale. The platform presented is based on longwave hyperspectral imaging of nearly 100 blocks of Manhattan, in addition to a geospatial radiosity model that describes the collective radiative heat exchange between multiple buildings. Despite assumptions in surface emissivity and thermal conductivity of buildings walls, the close comparison of temperatures derived from measurements and computations is promising. Results imply that the presented geospatial thermodynamic model of urban structures can enable accurate and high resolution analysis of instantaneous urban surface temperatures.

## Introduction

Cities are home to the majority of the world’s population and thus significantly determine global energy consumption, waste, and pollution. The dynamics of the urban energy budget, especially the thermal exchange between the densely built infrastructure and the surrounding environment, are not well understood. This is largely because the component of the energy budget associated with energy storage has been unattainable. The significance of this gap was highlighted in the early 1990’s through classic contributions by number of researchers working on the derivation of the urban energy budget. These included work on the urban heat island, radiative heat transfer and the stored energy^1–5^. This body of work was subsequently expanded to incorporate urban scale climate models that included the application of satellite remote sensing, resulting in better understanding of the thermal dynamic responses of the urban environments^6–16^. Nonetheless, the quantitative analysis of the thermal storage component is still elusive. This is because of the large number of unknowns in the urban space heat transfer equations. Time resolved analysis of the urban surface temperature is perhaps the most effective avenue for closing this knowledge gap. Advancing the understanding of the energy budget will lead to improvements in several areas: models of urban meteorology and air quality, models that forecast energy demand and consumption, technological innovations in building materials, heating and cooling technologies, as well as climate control systems and urban design. All of these improvements will enable energy efficiency at the building level and at city scale, while improving human health and the quality of the environment. When considering the state of art approaches to measuring urban surface temperatures, there have been number of challenges:

- Temperatures of vertical surfaces, which constitute the main portion of urban building surfaces, are inaccessible by satellite and/or aerial remote sensing. This information is essential in the accurate prediction of the urban storage flux.
- In the case of ground based remote sensing, the unknown surface emissivity can play a crucial role in the accurate determination of surface temperatures. Comprehensive databases of surface emissivity values for buildings in cities are not readily available.
- The Long Wave radiation measured by sensors is often a combination of gray body radiation of the target, as well as the radiation reflected (diffuse and specular) from other surfaces. Therefore, measured values do not always represent the intrinsic radiation of the target. Separation of the reflected portion is required for accurate assessment of the surface temperatures.
- The atmospheric constituents have significant effect on the interpretation of the measured values and the accuracy of numerical models when using actual values of sky radiance. While there are number of approaches to compensate for the atmospheric effects, with varying balance of complexity versus performance, the iterative approach (MODTRAN) was applied here.

The research presented in this article seeks to address some of these challenges. The measurements focus on vertical surfaces as a key element for derivation of multipath radiation, and estimation of the stored energy. We intentionally limit the scope of work to nighttime and corresponding stationary thermal radiation. This will help to obtain a well defined comparison between the model predictions and the measurements. We present results of studies performed in New York City, mapping the surface radiation from nearly 100 blocks of Manhattan’s West Side. This includes measurements using a hyperspectral imaging (HSI) instrument, and a theoretical model for radiative heat transfer. The model results are subsequently compared with the measured values.

This work benefits from a legacy of applications of spectroscopic imaging in earth sciences and remote sensing, including surface radiography and plume detection^17,18^. In the majority of those applications, imaging systems are deployed in a “downward-looking” configuration, mounted on moving platforms such as aircraft and satellites. In contrast, when considering urban energy research, stationary ground based imaging offers the advantage of persistence and a desirable field of view. Oblique view urban imaging has shown promise, for example for mapping the persistent leakage of refrigerant gases from a large number of structures in New York City^19^. Better understanding of the contribution of vertical surfaces can subsequently be integrated with global urban models, incorporating the effect of morphology.

A Long Wave Infrared (LWIR) imaging system manufactured by the Aerospace Corporation has been used in this study. It has a spectral range of 7.6–13.2 *μ*m and a spectral resolution of 40 nm. This spectral range also corresponds to the peaks of blackbody spectra for temperatures between 220 K and 380 K. The 1.1 mrad angular resolution of the instrument applied to target distances ranging from 1 to 5 km, corresponds to a spatial resolution of 1.1 m to 5.5 m per pixel, depending upon distance. The Doped Silica detectors result in Noise Equivalent Spectral Radiance (NESR) of 1 Flick (*μ*W cm^−2^ sr^−1^ *μ*m^−1^). They also allow gaseous compounds to be identified with high selectivity^19^.

## Results

**Figure 1:**
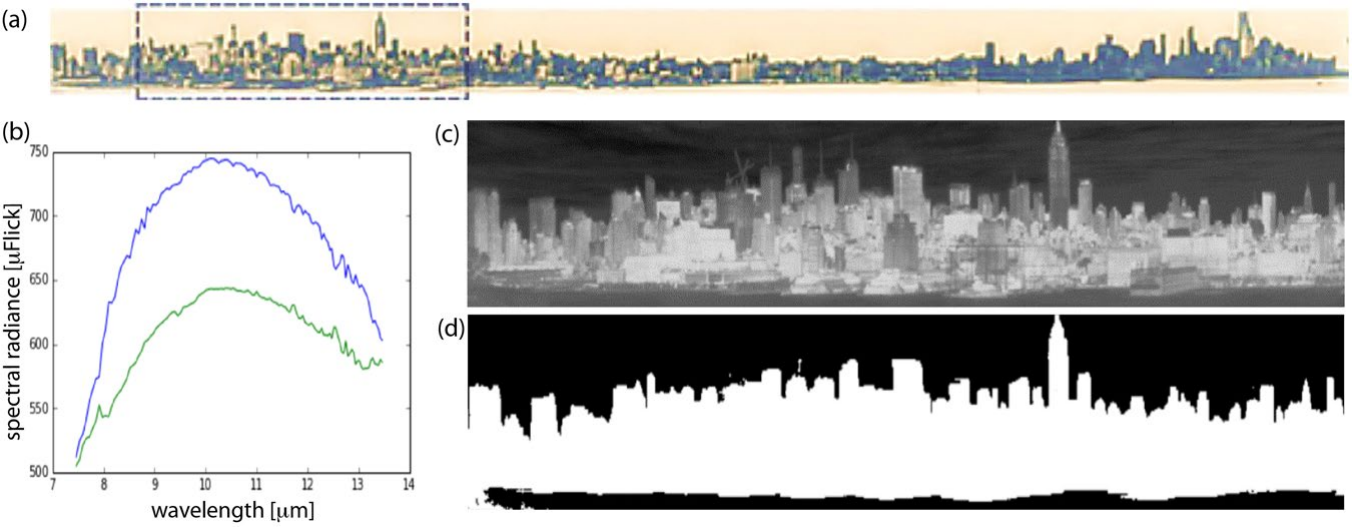
(**a**) Scan of eight kilometers along Manhattan West side (image by python https://codeshare.io/5ONkBP script, one wavelength from data cube). (**b**) Bi-cluster center spectrum. The blue curve represents building surfaces and the green curve represents sky and water. Wavelength in micrometers (horizontal) and radiance in micro flicks (vertical); (**c**) Brightness temperature image, averaged over all bands of hyperspectral data cube; (**d**) masked area of buildings.

**Figure 2:**
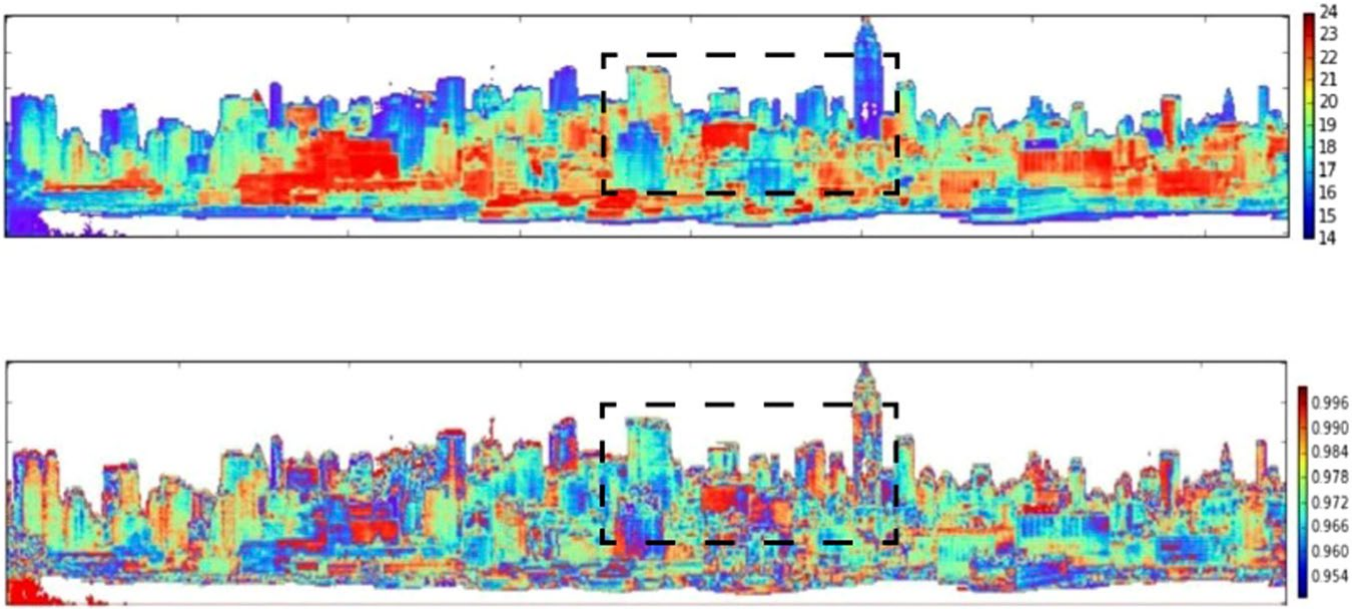
Map of temperature in °C (top), and emissivity (bottom) of the scene at 10 *μ*m wavelength. The framed region marks the area for which surface temperatures have been computed by the radiosity method.

**Figure 3:**
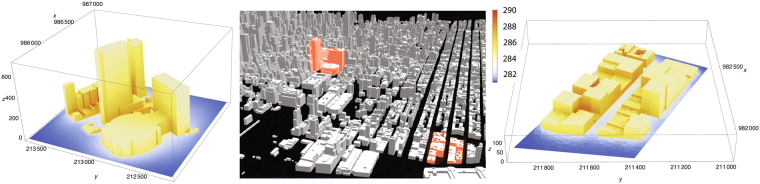
3D map of the area including modeled blocks of buildings (marked in red: 3 high rise blocks, upper left and 2 low rise, lower right) studied for model verification, and 3D visualization of computed surface temperatures (see text for details). Linear dimensions on the frame axes are in feet, temperatures in Kelvin.

Surface radiance measurements were carried out using a Long Wave hyperspectral instrument. Buildings were imaged along an eight kilometer portion of NYC’s West Side (Figs 1 and 2; *Data from the scene used for comparison with the surface temperature derived from the radiosity model are available upon request from the authors*). This was followed by a numerical simulation of Long Wave radiosity of the scene (Fig. 3). Measurements were carried out by installing the instrument on a rooftop vantage point at Stevens Institute of Technology in Hoboken, New Jersey. This provided an unobstructed view of Manhattan’s West Side with views including both low and high rise structures. Images were collected at 128 spectral bands, and for one week at cadences ranging from 10 seconds to 3 minutes. The variable cadence was used for the gas detection portion of the campaign, while only one time step was used for the instantaneous analysis of surface temperatures reported in this paper. The 1 to 5 meter spatial scale can enable the incorporation of useful attributes of buildings available in city databases (including fuel type, composition, occupancy, etc.), not used at this stage, but an option when the platform is at a higher level of maturity.

### Derivation of Surface Temperature and Emissivity from Hyperspectral Imagery

The spectral emission from a body is a function of wavelength, the body’s surface emissivity and its surface temperature. While the emissivity depends on the wavelength, the temperature is unique for an object at a given instance of time. A challenge in the application of thermographic imaging for mapping surface temperatures in heterogeneous terrains (such as buildings in a city) is that the surface emissivity is often unknown. That emissivity may be as low as 0.3 (aluminum and “low-e” glass) but mostly around 0.9 (concrete, brick, and polymeric composites). Therefore, the possible full range of emissivity values for building façades can correspond to as high as 30 °K temperature. Research using Short Wave infrared (SWIR) measurements have led to inventory of building surface properties^20^. This approach can be of great value if the SWIR and LWIR imagery are combined. In the absence of imagery in the lower wavelengths, and a database of building façade materials, we relied on in scene estimation of surface emissivity using LWIR imagery alone. An additional challenge is that the measured radiance at each pixel also includes effects of atmospheric scattering, absorption or emission by ambient gases along the path of observation. Therefore, when considering applications of thermography for studying urban thermodynamics, e.g. building energy and the urban heat island, significant errors may result if the emissivity effect is ignored. Hyperspectral imagery has been applied for the separation of the effect of emissivity versus surface temperature when considering measured radiance. These approaches have largely been applied to downward looking (aerial and satellite) remote sensing. Some techniques use calibrated curves of emissivity derived from laboratory experiments, in combination with instrumented measures of atmospheric parameters (e.g. using ASTER satellite^21,22^); others achieve the separation as a byproduct of the process for calculation of the atmospheric effects. Overall, calculation for the compensation of effect of the atmosphere is done following two distinct approaches: (1) using measured values of atmospheric constituents followed by modeling, the most commonly being the Moderate Resolution Atmospheric Transmission (MODTRAN)^23^, and (2) using radiance values measured by the hyperspectral sensor, without the use of auxiliary data, a technique commonly known as “In Scene Atmospheric Compensation” (ISAC)^24^. In this study we use the ISAC algorithm proposed by Young *et al*.^24^ for the calculation of the atmospheric effects, which also includes the separation of temperature versus emissivity. We also compared the ISAC approach with results obtained from the MODTRAN model which incorporates measured ambient concentrations of gases. Some background and brief summary of the approach and results are given below. In preparation for the application of ISAC algorithm following preprocessing was done^21,22^:

1. Conversion of raw telemetry data to engineering data
2. Radiometric and geometric calibrations
3. Bad pixel replacements
4. Spectral Smile removal

In contrast with the downward looking imaging, application of ISAC to the oblique-view, ground based imaging, should be considered with two caveats. (1) The first is the significance of the unequal distance from the sensor to the object, and the corresponding effect on the automated atmospheric compensation, which is inherent in the ISAC technique. In applications involving aerial or satellite platforms the object to sensor distance does not vary greatly, however in the oblique configuration, the distance can vary significantly. In our case (considering the scene in Fig. 1) it is in the range of 1 to 2 km. On the positive side, the smaller working distance in ground based sensing corresponds to a relatively smaller atmospheric effect. (2) The second point that needs to be considered is that ISAC relies on approximately 10% of the scene to be occupied by target emissivity close to 1. In downward looking remote sensing, these are often bodies of water which satisfy this condition. However, in the oblique view imaging, bodies of water (Hudson river in our case) are not ideal because of the ripple effect, observed in the oblique angle, diminishes the high emissivity advantage of water when applied to the ISAC algorithm. As a compromise, the large portion of high emissivity pixels corresponding to building materials composed of calcium (e.g. limestone), Silica (e.g. Bricks), or calcium silicates (e.g. concrete), with emissivity as high as 0.95, will to a great extent serve the black body requirement of the Algorithm. The error caused by this assumption was indirectly evaluated by spot checking the surface temperature calculations at selected pixels, using the MODTRAN model^23^, incorporating local measurement of atmospheric constituents^23^. The calculations were done by first masking the building region of the scene (Fig. 1) for the application of ISAC algorithms^24^, followed by the Temperature Emissivity Separation (Fig. 2). Masking was done using K-Means clustering with two clusters. One was the sky, clouds and water pixels as a cluster with lower temperature, i.e., lower radiance. The other are the buildings as a cluster being hotter thus higher radiance. The two cluster mean spectrum associated with the brightness temperature of the scene and the masked image are shown in Fig. 1.

Detailed calculation of atmospheric effects at selected pixel groups was carried out using the MODTRAN model^23^. The model incorporates the radiometric effects from the sky (e.g., reflection) as well as the prevailing atmospheric effects in the oblique view of the scene^25^. The model incorporated twenty-eight input parameters. Twenty-five of these were the atmospheric concentrations of various gases, twenty-two of which were taken from conditions typical in a mid-latitude summer air column (see Table 1), while H_2_O, O_3_, CO_2_ concentrations were measured ambient values obtained from local weather stations (Table 2). The remaining three parameters were air temperature, background surface temperature, and emissivity. Air temperature was obtained from local weather stations records. This approach follows the atmospheric correction procedures used for airborne HSI instruments such as ATLAS or HysPIRI, where radiosound launches are used to determine the gas concentrations. In our case the calculation of the background surface temperature requires the variable path length from sensor to the target location on the building surface. This was obtained using a 3D digital surface model (DSM) of the city derived from the NYC building data^26^. Other than the atmospheric effects noted above, the radiance recorded by the sensor also includes the sky radiance and scattered radiation along the observation path (known as down-welling radiation in airborne platforms). The calculation of this downwelling radiation is challenging in the case of oblique-view remote sensing. Nonetheless, certain assumptions can simplify the calculations. One is the use of similarity between the night-time radiation received by the objects in the scene, versus radiation received by the sensor from the sky. The building surfaces in the scene are vertically aligned and receive the sky radiance over a range of 90 degrees (from vertical to horizontal). The sensor swath width and height are 94 and 6 degrees, respectively. The field of view looking from the instrument, from Hoboken NJ, toward Manhattan is occupied by approximately 50% sky, 40% buildings and 10% river. The same can be said if the sensor was to be located in Manhattan looking toward the actual instrument location. Therefore, it can be assumed that the average of all pixels received in the 6 × 94 degree window is representative of what an object receives from its surroundings and seen by the sensor. This “in-scene” measurement was used to approximate the downwelling radiance.

**Table 1:** Concentration of trace gases used for modeling the atmospheric effect.

Compound	Concentration [g/cm^2^.m]
F11	2.93E-11
F12	5.03E-11
CCL3F	2.10E-19
CF4	2.10E-19
F22	1.26E-11
F113	3.98E-12
F114	2.51E-12
R115	2.10E-19
CLONO2	1.21E-12
HNO4	9.10E-14
CHCL2F	2.10E-19
CCL4	2.72E-11
N2O5	5.08E-17
CO	3.14E-08
CH4	3.56E-07
N2O	6.71E-08
O2	4.38E-02
NH3	1.05E-10
NO	6.29E-11
NO2	4.82E-12
SO2	6.29E-11
HNO3	1.05E-11

**Table 2:** Measured values used for modeling the atmospheric effect.

Compound	Concentration at Timestamp 04/13 15:22 EST
Water vapor	1.47 g/cm^3^
Ozone	0.00026 g/cm^3^
Carbon Dioxide	400 ppmv

(Weather station: NYC MetNet Station, 44th Street and Park Avenue, Latitude: 40.753081, Longitude: −73.976161).

The resulting values of surface temperature derived by MODTRAN were subsequently compared the ISAC results for selected pixels in a high rise area and a low rise part of the city (Fig. 3). Results of the comparison are quite good for this preliminary stage of work (Table 3). This comparison will need to be automated and applied to the entire scene in order to arrive at the statistics of the difference between the two approaches.

**Table 3:** Comparison of surface temperature derived using ISAC versus MODTRAN (in Kelvin).

Building Type	(ISAC)	(MODTRAN)
Low Rise Bldg	286.3	285.7
High Rise Bldg	284.1	283.3

### Comparison to Radiosity Model Predictions

In order to enable studies on the radiative heat transfer between urban structures (here buildings and streets) we have employed the radiosity method to compute the heat radiation emitted and reflected from all surfaces, including multiple reflections (see Methods). This method has been used in previous studies of radiative heat transfer in urban structures. Surface temperatures due to Long and Short Wavelength radiation have been estimated but only for simple block arrays that allowed for simplified computation of view factors^27^. Actual complex urban areas of a number of French cities have been studied recently by architects using also the radiosity method after a triangulation of the urban surfaces, see^28^ for a recent application of the approach. However, their precise implementation of view factor computation and surface temperature estimation techniques are not accessible, and computing time has not been published. A detailed comparison of this approach to a high resolution map of measured surface temperatures has not been performed. Hence it is important to establish a well documented, state-of-the-art approach (see Methods) that is experimentally verified. This is the main objective of this work.

Our model for the urban geometry is derived by surface triangulation from a geospatial dataset (see Methods). For each triangle of the surface mesh, we define its emissivity *ε*, wall thickness *d*, thermal conductivity *κ*, and the temperature *T* ^int^ on the inside of the surface wall. This information together with the Long Wavelength radiant flux *L* from the sky determines uniquely the outside surface temperatures in equilibrium for all triangular surface elements. Equilibrium refers to the assumption that there is no incoming radiant flux that varies over time, like day time solar radiation. Hence, we expect that our model can predict surface temperatures in the evening and during early morning before sunrise, or in strong cloud covered conditions. Without prior knowledge of the building envelopes composition and interior temperatures, we have assumed typical value of *ε*_wall,roof_ = 0.95, *κ*_wall,roof_ = 1.05 W/(K m), ${T}_{{\rm{wall}},{\rm{roof}}}^{{\rm{int}}}=293.15\,^\circ {\rm{K}}$ for walls and roofs, and *ε*_street_ = 0.93, *κ*_street_ = 1.25 W/(K m), ${T}_{{\rm{s}}{\rm{t}}{\rm{r}}{\rm{e}}{\rm{e}}{\rm{t}}}^{{\rm{i}}{\rm{n}}{\rm{t}}}=283.15{}^{^\circ }{\rm{K}}$ for streets and *d* = 0.2 m for all surfaces. Long Wavelength radiant flux from the sky was estimated to be *L* = 300 W/m^2^. These estimates are made as a baseline to establish a platform by which the radiant flux of the city can be analyzed at both the building level and the city level, simultaneously. Additional information on the building envelop materials would naturally improve the results.

Other studies based on radiosity and heat conduction across building walls sometimes use 0.1 m as a typical value for wall thickness^27^. For street surfaces typical values of the order of 0.3 m have been used^29^. While this is a reasonable range, one can expect a wider distribution of thicknesses in reality. It is expected that with increase of the availability of urban data, building information will also become more available in the near future, contributing to the model accuracy.

**Figure 4:**
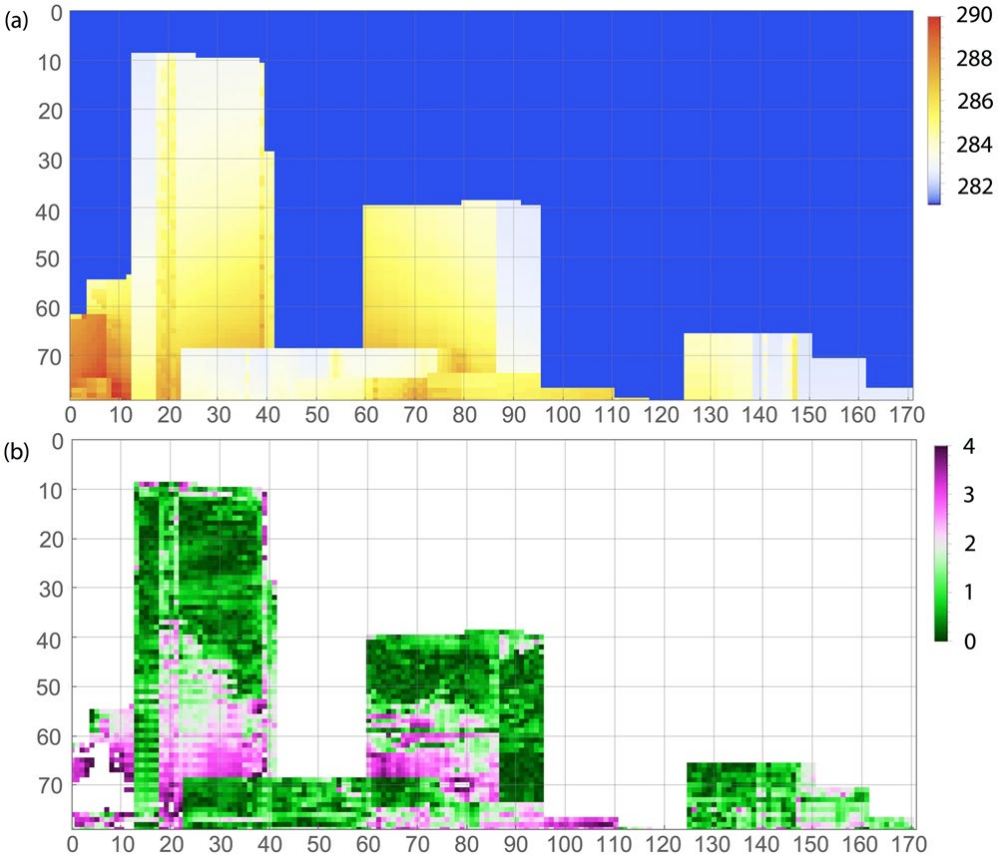
(**a**) Two-dimensional projection of computed surface temperatures for the two regions shown in Fig. 3. (**b**) Absolute temperature difference between (**a**) and experimentally determined surface temperatures, only for building surfaces. All temperatures are in Kelvin; the spatial dimensions are measured in camera pixels.

We compared the measured and model results for two block groups in Manhattan (Fig. 3), one is largely composed of high rise buildings (area HR) and the other composed of low rise buildings (area LR). Area HR consists of an area bounded by 7th to 8th Avenue and W 31st to 35th Street, and area LR consists of an area bounded by 10th to 11th Avenue and W 20th to 22nd Street. Figure 3 shows the isometric birds eye view of the blocks, and Fig. 4 is the virtual view of the same two block groups from the instrument location. The linear dimensions (in feet) of the two areas is indicated on the 3D views. A total number of 31339 triangular surface elements have been used to model the two areas. A 3D view of the resulting surface temperatures in shown in Fig. 3 for the two areas, with color coded temperature, and the same scale as in Fig. 4(a).

The 3D computed surface temperatures for the high rise and low rise areas is projected on the two-dimensional plane using the same view angle as the hyperspectral imager. It also has a pixel resolution that corresponds to the imager (1.1 rad per pixel in horizontal and vertical directions). Figure 4 shows the pixel matrix of the computed temperatures. Since the model describes only urban surfaces, the sky temperature is set to the average measured value. The HR and LR areas contain a large fraction of the building surfaces visible from the observation position. There are also surfaces of buildings between the two modeled areas that partially mask the buildings visible in Fig. 4(a). Computed and measured surface temperatures are compared by studying the absolute value of their difference. For the selected scene pixels visible by the imager, the agreement between the measured and computed values is very satisfying. Figure 4(b) shows that the lower bound of the differences is about 1 °K (for the majority of pixels) and that the larger deviations of about 3 °K are observed for the lower floors which have a greater number of multi path reflections.

**Figure 5:**
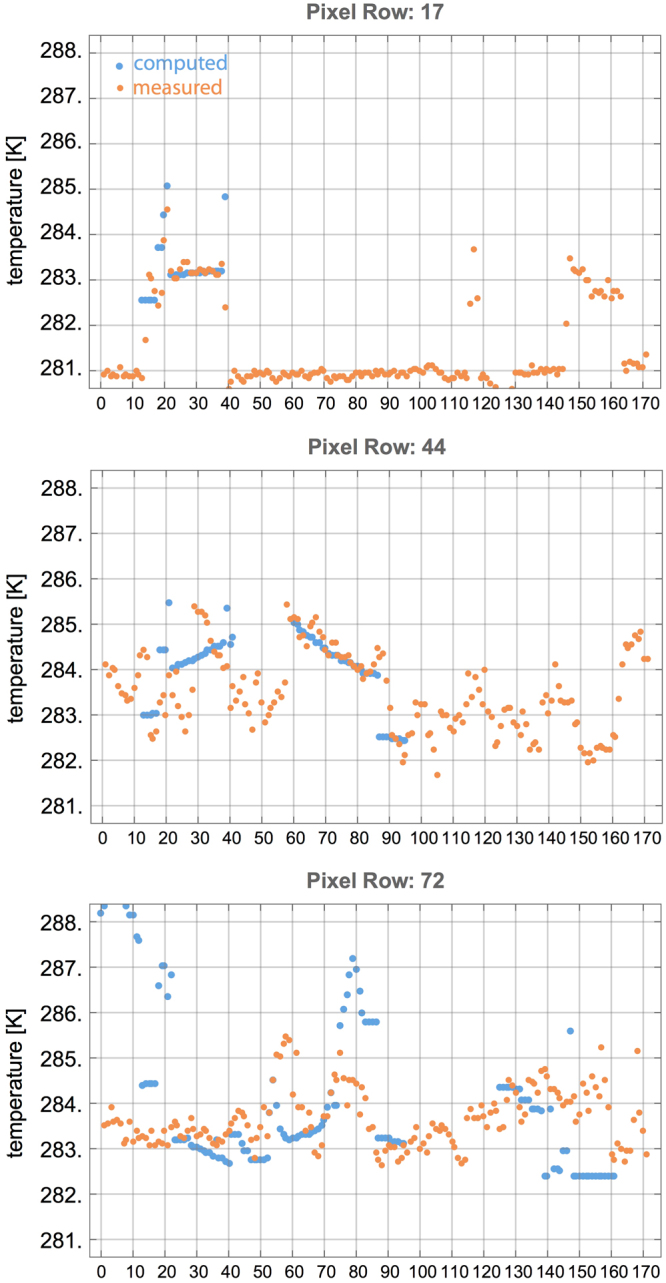
Measured and computed surface temperatures at three different elevation levels (pixel rows in Fig. 4), as function of camera pixels. Note that the measured data include also sky temperatures in the gap between buildings.

A more detailed comparison between computed temperatures and that derived from measurement is shown in Fig. 5. Three vertical elevations (camera pixel rows, cf. Fig. 4) are considered for comparison. Panel (a) corresponds to an elevation that cuts only the tallest building (between pixel columns 14 and 39). Measured and computed temperatures agree nicely, reproducing also the locally higher temperatures at one edge of the building, plausibly due to multiple reflections between facing walls (around pixel column 20). Panel (b) displays a cut through the second tallest building (between pixel columns 60 and 90). This building receives heat radiation from the tallest adjacent high rise building, producing the temperature gradient across its surface. Both measured absolute temperature values and the slope of the gradient are nicely reproduced by the computed temperatures. Finally, panel (c) displays a cut at a near ground elevation. While the temperatures show substantial spatial variations due to the combined effect of many low rise buildings, the overall range of the temperature profile shows reasonable agreement between measured and computed data. The discrepancy for the high rise between pixel columns 14 and 39 is caused by a masking building. We conclude that when compared to values derived from measurement, the model captures the main features of the radiative heat transfer in complex urban geometries, even when exact material parameters like emissivity and thermal conductivity are unknown, but replaced by typical values for urban materials.

## Discussion and Conclusions

At the introduction we highlighted the rational for mapping the vertical surface temperature in cities as a means for furthering the calculation of urban energy budget. The platform presented a number of considerations:

1. While the comparison between the experimentally derived and the modeled values for surface temperatures is convincing, the incorporation of the emissivity values derived from the measured radiance, or alternatively, values of emissivity from urban databases if available, can reduce errors caused by the constant emissivity assumption currently used in the radiosity model presented.
2. The spatial resolution of the experimentally measure radiance values is near the lower limit of accuracy for resolving building surface features. Higher resolution imagery will contribute greatly to enhancing the accuracy of surface emissivity and temperature. This can be achieved either by smaller instantaneous field of view (IFOV), or shorter distance to the target. The current spatial resolution of 1 to 3 meters introduces errors in certain parts of the scene when mixed pixel values are present (i.e. when a pixel represents more than one material, e.g. windows vs. facade). It should be added that we carried out a preliminary sensitivity test to study the effect with emissivity values including 0.85, 0.90, and 0.95. The difference between the modeled and measured values (similar to that shown in Fig. 4b) remained below 3 °K for these three emissivity values, although there were some differences in the distribution of surface temperatures across the building facades.
3. The measured values of radiance in such complex terrains is a combination of the direct radiation of a surface element and the radiation from other surfaces scattered from the target surface element. Using the radiosity model, it is possible to separate the two. We have not carried out this step; however, this can lead to a more accurate radiative transfer model of a city, particularly for application to building envelope energy performance and urban heat island studies. In addition, a time resolved study of heat radiation and surface temperatures can carried out.
4. The magnitude and distribution of surface temperature, as well as the differential between the surface temperature and air temperature is linked to convective turbulence at the near surface boundary. In other words, the sensible heat flux is effected by, and effects the turbulence. Despite the significance of this closed-loop process, the work presented here describes an approach for measurement and modeling of the Longwave urban radiation only. This can then be integrated as part of a comprehensive process (including convection) for modeling the urban energy balance.

## Methods

### Geospatial Dataset

Footprints of buildings in the form of two-dimensional polygons and the maximum building roof height above ground elevation were obtained from the shapefile (Name: “building_1015”) provided by the City of New York, Department of Information Technology and Telecommunications (https://data.cityofnewyork.us/Housing-Development/Building-Footprints/nqwf-w8eh). From these data polygons, the envelopes of connected buildings were constructed by removing internal walls between the buildings. The wall polygons obtained were divided along the vertical direction into smaller rectangles and finally triangulated together with the polygons for the roof and street surfaces to obtain a mesh of triangular surface elements. The radiosity method for computing radiative heat transfers has been applied to this mesh.

### Model for Radiative Heat Transfer and Surface Temperatures

The model has been developed and employed previously for patterned surfaces^30^. Urban geometry (building and street surfaces) is represented by a mesh of small surface “patches” given by *N* mutually joining triangles *P*_*j*_, *j* = 1, …, *N*, defined over a planar base plane (*xy*-plane). The triangles are oriented so that their surface normals ***n***_*j*_ are pointing to the outside of the buildings or towards the sky or streets. All surface normals are either normal or parallel to the base plane. Each triangle is further characterized by an emissivity *ε*_*j*_, surface thickness *d*_*j*_, and thermal conductivity *κ*_*j*_ described in the previous section. On the inside of the surface a local equilibrium interior temperature ${T}_{j}^{{\rm{int}}}$ is imposed for each triangle. We assume that the surface receives a homogeneous radiant flux *L* from the sky. The equilibrium temperatures *T*_*j*_ on the outside surfaces of the triangles are determined by equating the internal and external net flux densities for each triangle. The internal net flux is obtained from the stationary heat conduction equation ${q}_{j}^{{\rm{int}}}=-\kappa {\partial }_{n}{T}_{j}$ integrated across the surface thickness *d*_*j*_ yielding ${q}_{j}^{{\rm{int}}}=({T}_{j}-{T}_{j}^{{\rm{int}}}){\kappa }_{j}/{d}_{j}$. The external net flux ${q}_{j}^{{\rm{ext}}}$ is obtained as the sum of the incoming fluxes from the sky (*L*) and those scattered from all other visible triangles and the heat flux $\sigma {\varepsilon }_{j}{T}_{j}^{4}$ radiated by the surface *j* where *σ* is the Stefan-Boltzmann constant.

For the case of a single planar surface (*j* = *N* = 1), the condition ${q}_{1}^{{\rm{ext}}}={q}_{1}^{{\rm{int}}}$ yields

1 $$({T}_{{\rm{flat}}}-{T}^{{\rm{int}}})\frac{\kappa }{d}=\varepsilon (L-\sigma {T}_{{\rm{flat}}}^{4}),$$

which determines the outside surface temperature *T*_flat_ of the flat surface as function of known parameters.

For an urban geometry one has to consider multiple reflections between surface patches that contribute to the net external fluxes. To describe this effect, it is assumed that the surface patches are gray diffusive emitters, i.e., the emissivity is frequency independent and the radiation density is constant across the surface patches and emitted independent of direction. This is justified since the thermal wavelengths (microns) are small compared to urban structures and hence the size of the surface patches. We apply the radiosity method^31^ to obtain the external fluxes ${q}_{j}^{{\rm{ext}}}$. For a given surface patch *P*_*j*_, the outgoing radiant flux is given by the sum of emitted thermal radiation and the reflected incoming radiation,

2 $${J}_{j}=\sigma {\varepsilon }_{j}{T}_{j}^{4}+\mathrm{(1}-{\varepsilon }_{j}){E}_{j}$$

where we used that the reflectivity equals 1 − *α*_*j*_ for an opaque surface where *α*_*j*_ = *ε*_*j*_ is the absorptivity. How much energy two surface patches exchange via radiative heat transfer depends on their size, distance and relative orientation which are encoded in the so called view factor *F*_*ij*_ between patches *i* and *j*. *F*_*ij*_ is a purely geometric quantity and does not depend on the wavelength due to the assumption of diffusive surfaces above. It is defined by the surface integrals

3 $${F}_{ij}={\int }_{{A}_{i}}\,{\int }_{{A}_{j}}\,\frac{\cos \,{\theta }_{i}\,\cos \,{\theta }_{j}}{\pi {A}_{i}|{{\boldsymbol{r}}}_{ij}{|}^{2}}d{A}_{i}d{A}_{j}$$

where *θ*_*i*_ is the angle between the surface patch’s normal vector ***n***_*i*_ and the distance vector ***r***_*ij*_ which connects a point on patch *i* to a point on patch *j*, and *A*_*i*_ is the surface area of patch *i*. The view factor matrix obeys the important reciprocity relation *A*_*j*_*F*_*ji*_ = *A*_*i*_*F*_*ij*_ and additivity rule ${\sum }_{j}\,{F}_{ij}=1$. With this geometric quantity, the radiative flux received by surface patch *j* from all other surface patches can be expressed as ${E}_{j}={\sum }_{i}\,{F}_{ji}{J}_{i}$, and one can solve Eq. (2) for the vector of outgoing fluxes, yielding

4 $${\boldsymbol{J}}={[{\bf{1}}-({\bf{1}}-{\boldsymbol{\varepsilon }}){\boldsymbol{F}}]}^{-1}{{\boldsymbol{J}}}_{0},$$

where we combined the fluxes *J*_*j*_ from all patches into a vector ***J*** and the radiation $\sigma {\varepsilon }_{j}{T}_{j}^{4}$ into a vector **J**_0_ to use a matrix notation. Here **1** is the identity matrix and ***ε*** is the diagonal matrix with elements *ε*_*j*_. To obtain the surface temperatures *T*_*j*_ we need to compute the net heat transfer to surface patch *j* which is given by the incident radiation *E*_*j*_ minus the outgoing flux *J*_*j*_, leading to the net flux ${q}_{j}^{{\rm{ext}}}={\sum }_{i}\,{F}_{ji}{J}_{i}-{J}_{j}$. In vector notation this net flux becomes

5 $${{\boldsymbol{q}}}^{{\rm{ext}}}=({\boldsymbol{F}}-{\bf{1}})\,{[{\bf{1}}-({\bf{1}}-{\boldsymbol{\varepsilon }}){\boldsymbol{F}}]}^{-1}{{\boldsymbol{J}}}_{0}.$$

In the stationary state, the surface patch temperatures are then determined by the condition that the net external flux equals the net internal flux, ***q***^ext^ = ***q***^int^ where ***q***^int^ defines the vector with elements $({T}_{j}-{T}_{j}^{{\rm{int}}}){\kappa }_{j}/{d}_{j}$ due to heat conduction across the surface. This condition uniquely fixes the temperatures *T*_*j*_ when all other parameters including the flux *L* from the sky are known. In the following, technically, we include the sky as an additional surface so that we have now *N* + 1 surface patches. The corresponding additional matrix elements for the view factor matrix ***F*** follow from reciprocity and additivity rules, and we include the downward radiation *L* as the (*N* + 1)^th^ component in ***J***_0_.

### View Factors

The view factors *F*_*ij*_ between each pair of triangles of the surface mesh are computed using a modified version of the C program View3D which is freely available under GNU General Public License at https://github.com/jasondegraw/View3D. We have implemented a number of simplifications that result from the different classes of urban surfaces (walls, roofs, ground/streets) and employed a compact sparse matrix storage scheme that allows application of our method to geometries with a large number of surface mesh elements.

### Solving the radiosity equations

The numerical solution of the equations of the radiosity method follows these steps.

1. The triangular surface elements are grouped into three different classes: horizontal street patches (s), horizontal roof patches (r) and wall patches (w) that are perpendicular the base plane and connect the patches in class s and r.
2. Computing the view factors *F*_*ij*_. This needs to be done only for all patch class combinations (*w*, *s*), (*w*, *r*) and (*w*, *w*) with the restriction *i* < *j* for (*w*, *w*) since the view factors for *i* > *j* follow from reciprocity. The patches of classes *s* and *r* cannot see each other so that the view factor submatrix for these classes vanishes.
3. Constructing the total view factor matrix ***F*** for all triangles of classes *w*, *s* and *r* and the single enclosing surface describing the sky. This is done by using reciprocity to obtain the matrix elements for the patch class combinations (*s*, *w*) and (*r*, *w*). To obtain the view factor for the transfer from a surface patch *i* towards the sky we use the sum rule ${\sum }_{j}\,{F}_{ij}=1$, i.e., ${F}_{i{\rm{s}}{\rm{k}}{\rm{y}}}=1-{\sum }_{j\in \{s,r,w\}}\,{F}_{ij}$. The view factor for the transfer from the sky to a patch *i* follows from reciprocity as ${F}_{{\rm{s}}{\rm{k}}{\rm{y}}i}=\frac{{A}_{i}}{A}{F}_{i{\rm{s}}{\rm{k}}{\rm{y}}}$ where *A* is the total planar area of the urban area under consideration.
4. The inverse matrix of Eq. (5) can be computed as a truncated geometric series since the emissivities are sufficiently close to unity and the view factors *F*_*ij*_ < 1 with most of them in fact much smaller then unity. Hence the inverse kernel is given by ${{\boldsymbol{K}}}^{-1}\equiv {[{\bf{1}}-({\bf{1}}-{\boldsymbol{\varepsilon }}){\boldsymbol{F}}]}^{-1}={\sum }_{n=0}^{{n}_{c}}\,{{\boldsymbol{M}}}^{n}$ with ***M*** = (**1** − ***ε***)***F***. This expansion corresponds to a multiple scattering expansion for the heat radiation with *n* counting the number of scatterings. Only this expansion allows for a fast solution of the matrix equation for geometries with a large number of surface mesh elements. We find that *n*_*c*_ = 6 is sufficiently accurate approximation for the parameters used below.
5. Finally, we compute the surface patch temperatures *T*_*j*_ by an iterative solution of the equilibrium condition ***q***^ext^ = ***q***^int^ [see Eq. (5)] for given surface emissivity *ε*_*j*_, downward radiation *L*, interior temperatures ${T}_{j}^{{\rm{int}}}$ and effective thermal conductivity *κ*_*j*_/*d*_*j*_. The iteration steps are as follows:
  (i) Choose initial patch temperatures ${T}_{j}^{(\nu =\mathrm{0)}}$, e.g., the internal temperature. Convergence to the same result is obtained for a wide range of initial choices.
  (ii) Compute the external flux ${{\boldsymbol{q}}}^{{\rm{e}}{\rm{x}}{\rm{t}}(\nu =0)}=({\boldsymbol{F}}-{\bf{1}}){{\boldsymbol{K}}}^{-1}{{\boldsymbol{J}}}_{0}^{(\nu =0)}$ with the *N* + 1 dimensional initial vector ${{\boldsymbol{J}}}_{0}^{(\nu =\mathrm{0)}}=[L,\sigma {\varepsilon }_{1}{T}_{1}^{{(\nu =\mathrm{0)}}^{4}},\ldots ,\sigma {\varepsilon }_{N}{T}_{N}^{{(\nu =\mathrm{0)}}^{4}}]$.
  (iii) Compute the updated patch temperatures ${T}_{j}^{(\nu =\mathrm{1)}}$ from the equation ${q}_{j}^{{\rm{ext}}\,(\nu =\mathrm{0)}}=({T}_{j}^{(\nu =\mathrm{1)}}-{T}_{j}^{{\rm{int}}}){\kappa }_{j}/{d}_{j}$ for *j* = 1, …, *N*.
  (iv) Continue with step (i) to start the next iteration step, i.e., ${{\boldsymbol{q}}}^{{\rm{e}}{\rm{x}}{\rm{t}}(\nu =1)}=({\boldsymbol{F}}-{\bf{1}}){{\boldsymbol{K}}}^{-1}{{\boldsymbol{J}}}_{0}^{(\nu =1)}$ with the vector ${{\boldsymbol{J}}}_{0}^{(\nu =\mathrm{1)}}=\{L,\sigma {\varepsilon }_{1}{[({T}_{1}^{(\nu =\mathrm{1)}}+{T}_{1}^{(\nu =\mathrm{1)}}\mathrm{)/2]}}^{4},\ldots ,\sigma {\varepsilon }_{N}{[({T}_{N}^{(\nu =\mathrm{1)}}+{T}_{N}^{(\nu =\mathrm{1)}}\mathrm{)/2]}}^{4}\}$.

In (iv) and all following iteration steps it is useful to use the average of the last two iterations for the patch temperatures, as indicated here, to obtain rapid convergence. Typically, for the models and parameters used below, after about 20 iterations a stable solution for the patch temperatures had been reached (within a relative accuracy of 10^−4^).

### Data availability

The datasets generated during and/or analysed during the current study are available from the corresponding author on request.
